# Biochemical Assessment of Renal and Liver Function among Preeclamptics in Lagos Metropolis

**DOI:** 10.1155/2018/1594182

**Published:** 2018-07-31

**Authors:** Oloruntoba Ayodele Ekun, Oluwatumininu Mary Olawumi, Christian Chigozie Makwe, Nkeiruka Ogochukwu Ogidi

**Affiliations:** ^1^Department of Medical Laboratory Science, College of Medicine, University of Lagos, Nigeria; ^2^Department of Obstetrics and Gynaecology, College of Medicine, University of Lagos, Nigeria

## Abstract

**Objectives:**

Preeclampsia is a pregnancy specific syndrome. Studies have shown that preeclampsia has multiorgan dysfunction effects. This study evaluated biomarkers of renal and liver function among preeclamptic Nigerian women.

**Study Design:**

This was a cross-sectional study conducted among 49 preeclamptic women and 50 normotensive healthy pregnant women.

**Method:**

The baseline data comprising age, gestational age, and blood pressure were obtained. Venous blood and spot urine samples were collected from each participant. Plasma obtained from blood samples taken into lithium heparinized vacutainer bottles was assayed for electrolytes, urea, creatinine, total protein, albumin, and uric acid, while sera samples from blood samples taken into serum separation tube- (SST-) gel vacutainer were assayed for aspartate transaminase and alanine transaminase using ion selective electrode technique and Cobas autoanalyzer. Spot urine samples were assayed for protein and creatinine using Pyrogallol's reagent and Jaffe's methods, respectively. Microalbuminuria (protein/creatinine ratio) was generated from spot urine protein and creatinine data.

**Result:**

The plasma sodium, total protein, and albumin in preeclamptic group were significantly decreased (p<0.05) when compared with control. There was statistically significant increase (p<0.05) in microalbuminuria, plasma potassium, urea, creatinine, uric acid levels, serum AST, and ALT activities in preeclamptic group. A positive association (p<0.05) between alanine aminotransferase and biomarkers of renal function was observed.

**Conclusion:**

Preeclampsia has deleterious effects on renal and liver function as shown by alteration of these parameters.

## 1. Introduction

Preeclampsia has been described as a systemic syndrome of pregnancy, which is characterized by a new onset high blood pressure (systolic ≥140 mmHg or diastolic ≥90 mmHg) and proteinuria of ≥ 0.3 grams per 24 hours that occurs after 20 weeks of gestation in a woman that was previously normotensive [1]. It has been suggested to be a disease of the placenta, which possibly resulted from impaired trophoblast differentiation and invasion during early pregnancy. This impairment possibly stimulates sustained oxidative stress and a systemic inflammatory response in the affected women [2]. As recommended by the International Society for the Study of Hypertension in Pregnancy, the diagnosis of preeclampsia should requires blood pressures of 140/90 mmHg or higher on two occasions combined with either urinary protein excretion of ≥300mg/day or new onset of maternal organ dysfunction (creatinine ≥ 90*μ*mol/l), liver involvement (elevated transaminases), haematological complication (thrombocytopenia platelet <150,000/cmm), neurological complications, and fetal growth restriction [3]. Laboratory tests, such as liver function tests, quantification of urinary protein, or serum creatinine, may be helpful in characterizing the degree of end-organ damage, but none is specific for preeclampsia. It has been observed that preeclampsia has deleterious effects on maternal and perinatal health, particularly in the developing nations of the world [2]. It is a major complication of pregnancy [1]. It has been suggested that pregnant women with preeclampsia or eclampsia are likely to be at higher risk of end stage kidney disease and high blood pressure later in life [4, 5]. While most studies have been undertaken in high-income settings, some inconsistencies exist especially in developing settings where preeclampsia risk factors have been less explored [6].

A previous study suggested that women with severe preeclampsia possibly exhibits more prominent signs and symptoms of end-organ damage that may result in life-threatening disease [7]. Multiple organ systems may be affected in severe preeclampsia including dysfunction of the central nervous system (could lead to blurred vision, altered mental status, severe headache, and cerebrovascular accident), liver (which results in elevated serum transaminases), [3, 7] cardiovascular system (systolic blood pressure ≥160 mm Hg or diastolic ≥110 mm Hg), lungs (pulmonary edema, cyanosis), and/or kidneys [7]. Twenty-four hour urinary protein level is twice the normal limit in nonpregnant women [8].

Preeclampsia is more common in women with an underlying kidney disease. On the other hand, it has been suggested that preeclampsia itself increases the risk of kidney disease later in life [9]. It is a disease with worldwide significance to mothers and infants; it may have health hazards that increase maternal, fetal, and infant morbidity and mortality [8, 9]. It has been shown that preeclampsia has greatest impact in developing countries, where it accounts for 20-80% of strikingly increased maternal mortality, while in developed countries, preeclampsia has a major effect on fetuses and neonates [7–9].

## 2. Materials and Methods

### 2.1. Participants Selection and Study Design

A cross-sectional study was conducted on female preeclamptic patients attending Lagos University Teaching Hospital (LUTH), Ifako General Hospital, Isolo General Hospital, Lagos Island Maternity Hospital, and Amuwo Odofin Mother and Child Centre. A total of ninety-nine participants were recruited into this study, comprising 50 apparently healthy normotensive pregnant participants without urinary protein and 49 preeclamptic participants. This study was conducted between July and November 2017. Blood and spot urine samples were collected in the morning from all consenting participants, since proteinuria is known to exhibit circadian rhythm [10, 11]. The blood samples were collected into lithium heparinized and serum separation tube- (SST-) gel vacutainer bottles; the samples were centrifuged at 3,000 revolution per minute (rpm) for five minutes. Plasma and serum were extracted from lithium heparinized and SST-gel vacutainer, respectively. The samples were kept frozen at -20°C pending analysis.

### 2.2. Ethical Consideration

Approval was obtained from the Research and Ethics Committee of College of Medicine of University of Lagos (CMUL) and Lagos State Health Service Commission (HSC) prior to the commencement of the study. Informed consent was sought and obtained from each participant prior to the commencement of this study.

### 2.3. Inclusion Criteria

The inclusion criteria for this study include the following:

Apparently healthy pregnant volunteers with gestational age greater than 20 weeks, preeclamptic women with no previous history of hypertension and proteinuria before gestational age of 20 weeks and above.

### 2.4. Exclusion Criteria

The exclusion criteria for this study include the following:

pregnant women with other medical conditions such as viral hepatitis, diabetes mellitus renal failure, autoimmune diseases, anaemia, tuberculosis, chronic hypertension, and myeloproliferative disorders.

### 2.5. Methodology

Electrolytes were analyzed with SFRI 4000 ion selective electrode (ISE), while plasma urea, creatinine, uric acid, total protein, albumin, serum aspartate amino transferase, and alanine amino transferase were analyzed using Cobas C111 autoanalyzer by Roche. Spot urine creatinine and protein were determined using Biosystem BTS 350 semi-autoanalyzer by Biosystems SFA Pro.

### 2.6. Data Analysis

The data from the study was analyzed using SPSS version 21.0. Quantitative data were expressed as mean, standard deviation, and standard error of mean. Independent* t*-test was used to compare the groups. Pearson correlation coefficient was used to evaluate the degree of association. The level of significance for all the inferential statistics was set at p<0.05

## 3. Results

Table 1 presents the mean age, the gestational age, systolic pressure, and diastolic pressure of the participants in this study. There was a significant (p<0.05) increase in both the systolic and diastolic blood pressure among preeclamptic group. The comparative statistical analysis of plasma electrolytes, urea, creatinine, albumin, total protein, and uric acid among participants was presented in Table 2. There was a significant decrease (p<0.05) in the mean plasma sodium, total protein, and albumin among preeclamptic volunteers when compared with control group. A significant increase (p<0.05) in the mean plasma value of potassium, urea, creatinine, and uric acid as well as spot urine protein (albumin) creatinine ratio (microalbuminuria) among preeclamptic patients when compared with normotensive control (Table 2) was observed. There was a significant elevation (p<0.05) of transaminases in preeclamptic patients when compared with control (Table 3). A significantly higher plasma total protein and albumin (p<0.05) were observed among normotensive control group. The level of association of the parameters among preeclamptic group studied was evaluated (Table 4). There was a significant and positive association between the systolic and diastolic pressure (p<0.05) among preeclamptic women. Gestational age was observed to correlate positively with systolic and diastolic blood pressure (p<0.001, p = 0.01). A negative association was observed between plasma sodium and urea, sodium and creatinine (p=0.01, p< 0.001). There was a positive association between plasma potassium (p< 0.001) and creatinine, potassium and uric acid (p = 0.01), urea and uric acid as well as creatinine and urea (p<0.001).

## 4. Discussion

Preeclampsia has been a dreaded disease affecting women and their pregnancy for a long time. The disorder could be accompanied by possible numerous complications both to the woman and the unborn child. This has triggered phobia in some pregnant women and aroused the interest of obstetricians everywhere. It has been shown that development of preeclampsia involves a multifactorial process and multiorgan dysfunction with no individual factor strictly essential or sufficient for causing it [12].

In this study, there was no difference (p>0.05) in the mean age and gestational age of the preeclamptic test group when compared with the normotensive control. As observed in this study, there was a significant decrease (p<0.05) in the mean plasma sodium value among preeclamptic test group when compared with the control. This observation suggests that preeclampsia could be associated with hyponatremia. Our observation is corroborated by the previous study of Indumati et al. [13] who demonstrated a linear decrease in the mean sodium value among preeclamptic volunteers when compared with the normal pregnancy. Available data suggests that preeclamptic women with hyponatremia may present with severe symptoms [14]. Thus hyponatremia in patients with preeclampsia may be associated with increased risk of maternal seizures [15]. Razavi et al. [16] showed that hyponatremia tends to occur more frequently among preeclamptic women with severe disease. Our finding regarding plasma sodium agreed with the previous studies of Razavi et al. [16] and Dwivedi et al. [17]. While the mechanism of hyponatremia in preeclampsia is unclear, it has been suggested, however, that syndrome of inappropriate antidiuretic hormone secretion (SIADH) and low effective plasma volume which possibly leads to a nonosmotic release of ADH may be involved in the development of hyponatremia in preeclamptic patients [18].

Furthermore, there was a significant increase in the mean plasma potassium level of the preeclamptic volunteers in this study when compared with the normotensive pregnant control. Our observation with regard to potassium is in consonance with the previous study of Dwivedi et al. [16]. In preeclampsia, it has been reported that a decrease glomerular filtration rate (GFR) and renal plasma flow (RPF) possibly decreases sodium delivery to the distal nephron as well as causing decreased potassium secretion [19]; thus suggesting the possibility of inhibition of urinary potassium excretion prior to administration of magnesium in some preeclamptic women [19]. An increase in the mean plasma potassium as observed in preeclamptic patients may also possibly be secondary to the effect of magnesium sulphate infusion therapy as Hudali and Takkar [20] as well as Iglesias et al. [21] reported hypocalcaemia and hyperkalemia during magnesium infusion therapy in preeclamptic patients. It is thought that administration of magnesium in the treatment of preeclampsia possibly reduces the activity of plasma renin and angiotensin converting enzymes resulting in low levels of renin, angiotensinogen, angiotensin II, and aldosterone [19, 22, 23]. Thus magnesium infusion possibly increases sodium and chloride excretion but suppresses potassium excretion by the kidney [24, 25]. An increase in potassium value as observed in our study was similar to the previous finding by Handwerker et al. [26] where a significant increase in ionized magnesium and potassium was observed among preeclamptic women. On the other hand, Adewolu [27] showed no significant difference between the mean plasma potassium values in preeclampsia and normal pregnancy.

Also, our study showed that the mean plasma urea, creatinine, and uric acid levels were significantly raised (p<0.05) among preeclamptic population when compared with normotensive control pregnant women. Our observation was similar to the previous study by Vyakaranm et al. [28] that reported an increase in the mean uric acid and creatinine values among preeclamptic patients. Previous study has shown that blood urea nitrogen (BUN) and creatinine levels among preeclamptic women are similar to the levels seen in nonpregnant women because of reduced glomerular filtration rate (GFR) and reduced renal plasma flow (RPF) [11] as against normal pregnancy that is characterized by increase in GFR [29, 30] as well as RPF. Thus elevated levels of creatinine may be due to decrease urinary clearance secondary to reduced GFR and increased reabsorption [31]. A significant increase in the mean uric acid level as seen among preeclamptic volunteers in this study is in consonance with previous studies by Salako. et al. [32] and Mert et al. [33] that indicated a significant elevation in serum uric acid in preeclampsia test group when compared with normotensive control.

Also, it has been shown that uric acid concentration falls initially by 25% to 35% in normal pregnancy partly due to estrogen, expanded blood volume, and increased glomerular filtration rate [34]. However, it later rises to a nonpregnant women level at term. However, in preeclamptic patients, uric acid tends to increase much earlier than the onset of hypertension and proteinuria [35] and fall in GFR [36]. Thus elevated uric acid as observed in this study may suggest an antioxidant effect in response to oxidative stress [37]; this may be protective on one hand; however it has been shown that hyperuricemia elicits proinflammatory effect which may lead to endothelia dysfunction [38] possibly causing vascular damage and hypertension [39, 40], thus worsening the outcome. Recent studies have suggested an association between serum uric acid in preeclampsia and the severity of the disease [41, 42]. Thus increased renal function biomarkers parameters as observed among preeclamptic group in this study are in consonance with previous study by Manjareeka and Nanda [43] whereas Salako et al. [32] reported no significant difference in creatinine level of preeclampsia and normotensive pregnant women.

Moreover, the mean plasma total protein and albumin as observed in this study were significantly (p<0.05) lower among preeclamptic group when compared with normotensive control group. This could possibly be a consequence of urinary loss of protein as observed in this study with spot urine protein which was significantly higher (p<0.05) among preeclamptic test group when compared with the normotensive control. It has been shown that proteinuria as seen in preeclampsia might be the consequence of a loss of both size and charge selectivity of the glomerulus [44]. Hence Muller-Deile and Schiffer [11] reported that a common reason for proteinuria in pregnancy is preeclampsia. In this study, there were significantly raised (p<0.05) spot urinary protein (albumin) and urine protein creatinine ratio (UPC) among preeclamptic patients when compared with the normotensive control volunteers. Previous study has shown that accuracy of UPC ratio in predicting 300mg protein in 24-h urine collection in pregnant patients with suspected preeclampsia had a sensitivity of 90%–99% and specificity of 33%–65% [11, 45]. In addition, Durnward et al. [46] suggested that UPC ratio can predict severe preeclampsia and can be used for rapid diagnosis of severe preeclampsia as the correlation of UPC ratio and 24-hr proteinuria increases with the amount of proteinuria. It appears that a significant increase in albumin-creatinine ratio (microalbuminuria) among preeclamptic group could possibly be an indication of renal pathology, an observation which may support the multiorgan dysfunction effect of preeclampsia as a disease.

Furthermore, we observed that transaminases (ALT and AST) were significantly elevated among preeclamptic women (p<0.05). Our observation is similar to the previous studies by Hazari et al. [47] and Dacaj et al. [48]. It is thought that elevated transaminases among preeclamptic women are possibly due to hypoxic effect of preeclampsia on their livers, since hypoxia results in necrosis with a resultant degeneration of hepatocytes [48].

An assessment of the degree of association among the parameters studied in preeclampsia shows that gestational ageing is positively (p<0.05) associated with increase in both systolic and diastolic blood pressures. There were positive correlations (p<0.05) between urea and creatinine, total protein and albumin, and potassium and creatinine. Positive associations (p<0.05) were also observed between uric acid and urea, as well as between alanine aminotransferase and biomarkers of renal function (uric acid, urea, and creatinine). On the other hand, negative association existed between uric acid and albumin, as well as between albumin and urea. The positive association between alanine aminotransferase and markers of renal function suggests that preeclampsia is possibly associated with increased risk of hepatic and renal pathology.

## 5. Conclusion

Our study shows that preeclampsia has deleterious effects on renal and liver functions as shown by deraignment in biomarkers of renal and liver function with respect to normotensive pregnancies. Preeclampsia results in significant electrolyte imbalance. Thus there is a need for close monitoring of women with preeclampsia.

## Figures and Tables

**Table 1:** Baseline characteristics of the studied participants.

Variable	Preeclampsia mean ±SD (n=49)	Control mean ±SD (n=50)	t value	p value
Age (year)	33.18 ± 4.55	32.44 ± 4.99	-0.77	0.44
Gestational Age (weeks)	29.45 ± 3.03	29.80 ± 4.02	0.49	0.63
Systolic BP (mmHg)	172.55 ± 24.16	113.02 ± 9.95	-16.09	<0.0001*∗*
Diastolic BP (mmHg)	112.47 ± 17.73	70.20 ± 9.09	-14.97	<0.0001*∗*

*∗*Significant probability (probability <0.05), BP: blood pressure.

**Table 2:** Comparative analysis of renal function parameters in preeclamptic and normotensive participants.

Variable	Preeclampsia mean ±SD (n=49)	Control mean ±SD (n=50)	t value	p value
Sodium (mmol/L)	130.39 ± 6.30	133.16 ± 6.75	2.11	0.04*∗*
Chloride (mmol/L)	104.89 ± 6.75	105.62 ± 3.58	0.69	0.50
Potassium (mmol/L)	4.83 ± 1.08	4.14 ± 0.52	-4.05	<0.0001*∗*
Bicarbonate (mmol/L)	15.73 ± 3.73	17.10 ± 3.41	1.90	0.06
Plasma Urea (mmol/L)	3.80 ± 2.09	1.26 ± 0.66	-8.21	< 0.0001*∗*
Plasma Creatinine (*μ*mol/L)	93.82 ± 38.39	45.57 ± 11.20	-8.53	<0.0001*∗*
Uric Acid (*μ*mol/L)	398.24 ± 160.36	213.66 ± 50.00	-7.76	<0.0001*∗*
Spot urine Creat (mg/L)	316.19 ± 205.24	222.06 ± 130.69	-2.73	0.01*∗*
Spot urine Prot (mg/L)	1666.24 ± 1188.26	512.12 ± 343.13	-6.59	<0.0001*∗*
Albumin/creatinine (*μ*g/mg)	632.38 ± 527.09	246.25 ± 82.26	-5.12	<0.0001*∗*

*∗* Significant probability (probability <0.05).

**Table 3:** Comparison of liver function parameters in preeclampsia and control.

Analytes	Preeclampsia (n=49)	Control (n=50)	t-value	p-value
	mean ± SEM	mean ± SEM		
Plasma T. Prot (g/L)	49.55 ± 7.82	57.80 ± 7.28	5.44	<0.0001*∗*
Albumin (g/L)	23.10 ± 3.76	32.42 ± 5.22	10.18	<0.0001*∗*
AST (U/L)	112 ± 18.61	16.83 ± 2.58	-5.14	<0.0001*∗*
ALT (U/L)	31.75 ± 4.63	4.78 ± 0.42	-5.86	<0.0001*∗*

*∗* Significant probability (probability <0.05).

**Table 4:** Degree of association between parameters studied among preeclamptic group.

Variable	Correlation coefficient “r”	p value
Gestational age vs systolic BP	0.42	<0.001 *∗*
Gestational age vs Diastolic BP	0.35	0.010*∗*
Potassium vs Creatinine	0.43	<0.001*∗*
Potassium vs Uric acid	0.39	0.010*∗*
Sodium vs Urea	-0.35	0.01*∗*
Sodium vs Creatinine	-0.58	< 0.001*∗*
Urea vs Creatinine	0.46	<0.001*∗*
Urea vs Albumin	-0.41	<0.001*∗*
Urea vs Uric Acid	0.71	<0.001*∗*
Uric Acid vs Creatinine	0.53	<0.001*∗*
Uric Acid vs Albumin	-0.29	0.040*∗*
ALT vs Uric Acid	0.500	<0.001*∗*
ALT vs Urea	0.455	0.001*∗*
ALT vs Creatinine	0.589	<0.001*∗*

## Data Availability

The data used to support the findings of this study are available from the corresponding author upon request.
